# Application of clove and dill oils as an alternative of salphos for chickpea food seed storage

**DOI:** 10.1038/s41598-021-89936-4

**Published:** 2021-05-17

**Authors:** Narendra Kumar, S. M. Paul Khurana, Vashist N. Pandey

**Affiliations:** 1 Amity Institute of Biotechnology, Amity University Haryana, Gurgaon, Manesar, Haryana 122413 India; 2 Experimental Botany and Nutraceutical Lab, Department of Botany, DDU Gorakhpur University, Gorakhpur, U.P. 273009 India

**Keywords:** Biological techniques, Plant sciences

## Abstract

Mycological investigations of 25 samples of stored chickpea food seeds (*Cicer arietinum* L.) from grocery stores of Gurgaon and Gorakhpur revealed occurrence of seventeen fungal species belonging to genus viz., *Alternaria, Aspergillus, Chaetomium, Colletotrichum, Curvularia, Fusarium, Penicillium, Rhizopus, Rhizoctonia,* and *Sclerotium*. In these *Aspergillus flavus, A. niger, Fusarium oxysporum* had dominance in terms of per cent occurrence. Only one species of Bruchid (*Callosobruchus chinensis* L.) *occurred* in all the 25 samples. The biodeterioration of seeds inoculated with fungi: *A. flavus, A. niger, F. oxysporum* and the insect—*C. chinensis*, revealed their role in seed deterioration. For chickpea food seed protection essential oils *were* extracted from edible commodity(clove(Lavang and dill(sowa) leaf). Clove(Lavang) oil registered highest antifungal activity inhibiting (100%) mycelial growth of fungi, viz. species *Aspergillus flavus, A. niger, Fusarium oxysporum* at 300 ppm but was fungicidal at 400 ppm. Dill (Sowa) oil showed complete inhibition at 400 ppm and was fungicidal at 500 ppm. While mixture of both the oils (clove and dill) showed complete inhibition (100%) and fungicidal action at 400 ppm against the dominant fungi. The oils showed 100% insect repellent activity and were found fungicidal at 0.02 ml dose and also insecticidal. The mixture of oils was cidal at 0.02 ml dose. The mixture of oils showed a broad antifungal spectrum at 500 ppm while only 70–93% inhibitory activity at 300 ppm. The oils' mixture's activity was not affected by temp, storage and autoclaving up to 150 days. Oils physico-chemical properties were studied. GC–MS analysis of clove(Lavang) oil depicted major components: 75.63%eugenol while dill(sowa) leaf oil had 25.14% apiole. Formulation of Mixture of oils was more effective showing complete seed protection i.e.no growth of fungi and insects upto 150 days storage than salphos (150 days). While salphos controlled only maximum three fungi (*A. terreus, C. dematium, F. moniliforme*). The formulated oils mixture did not have any adverse effect on the chickpea seeds and increased *their* shelf life.

## Introduction

The chickpea or chick pea (*Cicer arietinum*) is the most important and versatile legume, from Fabaceae, subfamily Faboideae. It is locally known as gram or Bengal gram or garbanzo. This has high nutritional value. Dried chickpea seeds have 61% carbohydrate, (17–22%) proteins and 6% fat by weight. It is one of the earliest cultivated legumes. Its history is 7500-year-old, as the remains have been found in the Middle East. Chickpea is a key ingredient in hummus and chana masala. This is ground into flour to make falafel. It is also used in salads, soups and stews, curry and other food items like roasted/baked channa.

Chickpea is typically stored for 6–8 months after harvest. But more than 90% of farmers do not take any precautions to protect it. Its proper storage enables farmers to earn high profit margins. Most farmers sell their chick pea seeds in village/local and urban markets. Aprox. 20–30% of the stored chick pea food seeds get deteriorated by fungi and insects. Then on scientific storage of chick pea in rural areas leads to heavy losses of chickpea by fungi and insects. But detailed studies on such deterioration of stored chickpea food seeds have not been made so far. The chickpea seeds in the storage condition become more susceptible to fungal infection resulting in the lowering in seed germination and deterioration in storage. A damaged seeds produce abnormal seedlings. Thus farmers are adviced to use pathogen free healthy seeds to overcome the losses in productivity. But without testing for seed health, it is not possible to detect % healthy seeds to ensure better productivity.

A seed borne pathogen whether present internally or externally or associated with the seed as contaminant may result seed rot, seed necrosis, seed abortion, reduction or elimination of germination capacity thereby affecting of seed quality. This also causes seedling damage which produce crop diseases at later stages of plant growth through local or systemic infection^1^. The base of total food grain production in a country depends much on quality of the seeds. Seed health has an important role in successful cultivation and yield exploitation of a crop species. Some synthetic fungitoxic chemicals are used to control mycotoxin producing fungal infection but they also have their side effects^2,3^.

The synthetic preservatives because of carcinogenic effects are getting lesser preference so alternate for farmers is to shift on aromatic plants having of essential oil which have potent pesticidal potential. So potential of mixture essential oils is the need of time as single oil is not enough to protect pests. So if exploiting synergies of several compounds may be a solution to handle this problem. But little is known on interactions that leads to additive, synergistic or even antagonistic effects^4^.

Lavang(clove) botanically known as *Syzygium aromaticum* L. are flower buds of aromatic nature. This have large leaves bearing of crimson flowers can be grouped in terminal clusters. This reaches up to 8–12 m. Flower buds initially looks a pale hue turning gradually in green, then to bright red when ready for harvest. Chemically Clove have eugenol, eugenyl acetate, caryophyllene α-humulene, β-elemene, ledol and α-cadinene^5^.

Dill botanically called as *Anethum graveolens* L locally known as sowais. This is an annual herb used as vegetable belongs to celery family Apiaceaeused. This grows up to 40–60 cm having of slender hollow stems. Entire herb including stem, leaf and fruit are may be considered for flavoring of food and beverages. Used in cosmetics and in various medicinal preparations. This have antimicrobial, anti-oxidative and antispasmodic activities^6,7^. Sowa leaf contains o-cymene, apiole, α-thujene, exo-2-hydroxycineol, limonene, β-phellandrene dithydroumbellulone and 3-isoproyl-4-methyl-1-pentyn-3-ol myristicine^8^.

So mycological analysis of stored chickpea food seeds were conducted in order to find out thedominant fungal species and insects responsible for its biodeterioration. The essential oil extracted from Lavang and sowa leaf locally available was evaluated for its fugitoxicity and repellent activity. Its chemical analysis were done. Experiments conducted for its MIC, nature, spectrum and storage. The efficacy of mixed oils (Lavang(clove) and dill(sowa) leaf) was compared with commercial pesticides Salphos for chickpea food seed protection up to150 days of storage.

## Materials and methods

### Collection of chickpea food seed samples and mycological analysis

Samples of chickpea food seed kept in storage (6–8 months) of were collected from 25 sites (10 grocery stores + 15 farmer’s stores) of Gorakhpur (UP) and Gurgaon (Haryana) in presterilized polyethylene bags of 1 l. The mycological flora identification analyses of chickpea food seeds were conducted both through agar plate^9^ using Czapek dox agar medium (each containing15ml medium) and standard blotter^10^ techniques.

Composition of Czapek dox agar medium Ingredients Gms/L (Sucrose 30.00; Sodium nitrate 2.00; Dipotassium phosphate 1.00; Magnesium sulphate 0.50; Potassium chloride 0.50; Ferrous sulphate 0.01; Agar 15.00;Final pH (at 25 °C) 7.3 ± 0.2. About 100 seeds were equidistantly placed on Czapeks dox agar medium in separate Petri plates in agar plate technique. Each contained 5 seeds. In blotter test, seeds were similarly plated on three layered moistened blotter pads in sterilized Petriplates. These assay plates were then incubated at 25 ± 2 °C and observed daily from 7-to-15 days for observing growth of fungal colonies. Fungal colonies on the seeds were isolated and subcultured. The microscopic and macroscopic observations were taken and identified with the help of available literature^11–14^ as well as by comparing their morphological and cultural characteristics with authentic cultures maintained in Mycology Lab, Dept of Botany, University of Gorakhpur and AIB, Amity University Haryana. For detecting the internal seed borne pathogens, chickpea seeds were first surface sterilized with aqueous 0.1% mercuric chloride for 2 min. They were then washed with double distilled water and then subjected to agar plate and standard blotter techniques for isolation of the fungi. Each fungal species (%) associated with seeds samples of chickpea was estimated.

### Culturing insect-***Callosobruchus chinensis*** (L.)

This was done from infested stored chickpea seeds collected from twenty five places of Uttar Pradesh, India and identified^15,16^. This was maintained in newly harvested chickpea seeds incubated at laboratory (28 ± 2 °C temperature) in darkness to obtained same aged insects. No insecticide treatment was given.

### Evaluation of deterioration caused by dominant fungi and insect

For studying effect of dominant fungal species viz., *Aspergillus flavus, A. niger, Fusarium oxysporum* causing deterioration parameters viz., respect to weight loss, seed germination, carbohydrate and protein content was evaluated. This was conducted by taking local variety of freshly harvested sterilized chickpea seeds in containers of presterilized polyetylene bags that was having of (250 g seeds/bag). This was inoculated by two discs (7 mm diam) of these dominant different fungal species separately. Similarily 6 insect—*Callosobruchus chinensis* were taken and inoculated in presterilized polyetylene bags separately. The insect inoculated chickpea seed samples were incubated for storage up to 21 days in laboratory conditions at room temperature. The Experiments were conducted twice and each trial was having five replicates.

The deterioration caused by fungi/insect in terms of carbohydrate content in chickpea seed were studied following Anthrone method^17^. The Carbohydrates were dehydrated through Conc. H2SO4 for forming furfural. Furfural then condenses with anthrone (10-Keto-9, 10 dihydro anthracene) to form a blue-green coloured complex. This was measured through calorimeter at 630 nm. The protein content estimation was done following Lowry et al*.*^18^ by taking bovine serum albumin as standard. The optical density of each chickpea seed sample was taken at 650 nm.

### Extraction of essential oil from Lavang-*Syzygiumaromaticum* (Myrtaceae) and leaves of sowa (*Anethum graveolens* L. (dill) and Fumigant fungitoxicity testing against fungi

The essential oil was extracted from 200 g dried and ground Lavang(clove), and 1000 g chopped leaves of dill(sowa) (*Anethum gravelens*) through hydrodistillation in 2 L water in Clevenger’s apparatus at 90 ± 2 °C up to 5–9 h separately. The isolated essential oils were dried over anhydrous sodium sulphate separately. This was stored at 4 °C under sterile condition for further use. For developing fungitoxic fumigant activity of clove buds-*S. aromaticum* (Myrtaceae) and leaves of sowa (*Anethum graveolens* L. (dill) Apiaceae against fungal species were evaluated separately through Inverted petri plate technique^19^ at 500 ppm (0.025 ml).

### Physiochemical properties of Lavang and sowa oil

The oil was characterized by determination of Specific gravity, Refractive index, Optical rotation, Acid value, Ester value, Saponification value and Solubility characteristic following the methods of Langenau^20^.

### GC–MS investigations

Here required amount (0.1 μl) of essential oil of Lavang and dill leaf were taken for analysis of GC and GC–MS analysis separately. The GC was framed of an Agilent Technology 6890 N gas chromatograph data handling system. This having equipped by a split-splitless injector. The split ratio taken was 50:1. This fitted with a FID (flame ionization detector) by using N2 being a carrier gas that was in a flow rate 1 ml min^−1^. The column taken was (30 m × 0.32 mm, 0.25 μm film thickness) HP-5 capillary column. The temperature taken for the study was: starting temperature of 60 °C (hold—2 min) programmed at a rate of 3 °C · min^−1^ to a final temperature of 220 °C (hold—5 min). Temperatures of the injector and flame ionization detector (FID) were kept at 210 °C and 250 °C respectively. The identification of Compound was carried out by calculating their Kovats indices^21^ and by doing comparison of mass spectra which is reported in the literature^22^ and that is present in database of GC–MS computer (NIST 98 and Wiley-5).

### Fungitoxic and insect repellent investigations of essential oil of Lavang and sowa leaf

The minimum inhibitory concentration (MIC) of Lavang and sowa leaf essential oil was investigated following poisoned food technique^23^. Here various concentration of the Lavang and sowa leaf essential oil which covered from 200 to 800 ppm were prepared. This was done through dissolving requisite amount of oil in 0.5 ml acetone and then mixing with 9.5 ml Czapeks-dox agar medium separately. For control sets Petridishes with acetone and medium not having of Lavang and sowa leaf essential oil were taken. The Fungal discs of dominant fungal species (5 mm diam) which was taken from 7 days old culture (periphery region) of each of test fungi viz., *Aspergillus flavus, A. niger, Fusarium oxysporum* were inoculated aseptically at treatment and control sets each separately. All the sets were kept for 6 days at 28 ± 2 °C. The Diameters of fungal colony of control and treatment sets of *Aspergillus flavus, A. niger, Fusarium oxysporum* were taken separately in mutually perpendicular directions on the 7th day. The Experiments were done twice. This was having of 3 replicates. The repellent activity of the essential oil of Lavang (clove) and sowa (dill) leaf against insect-*Callosobruchus chinensis* was done following the method of Tripathi and Kumar^24^ with slight modification.

To find out nature of antifungal properties of the Lavang and sowa leaf oil treated fungal disc of *Aspergillus flavus, A. niger, Fusarium oxysporum* that was showing complete inhibition of mycelial growth upto 7th day were taken. This were washed with sterile water. This was placed again on fresh solidified medium for taking an observation of revival of mycelial growth. The fungitoxic spectrum of essential oil of Lavang, sowa leaf and mixture oil (1:1 ratio) were investigated on various fungi isolated from seeds samples of chickpea. The effect of autoclaving and storage on the fungitoxicity of Lavang and sowa leaf oil and mixture oil was determined^25^. Each experiment was repeated twice and had 3 replicates.

### Formulation of mixture oil (*Syzygium aromaticum* (Myrtaceae) and leaves of sowa (*Anethum graveolens* L.) and Comparison of efficacy with synthetic fumigant-Salphos

As the cost of 100 g Lavang is Rs299 which is high for farmers and cost of sowa leaf is Rs.10–15/kg (even free in our farmers field), so for developing low cost formulation mixture of Lavang and sowa leaf oil in 1:5 ratio was prepared. For this 1 ml of Lavang oil was taken in clean (ed) sterilized test tubes and added 5 ml of sowa leaf essential oil.

**Figure 1:**
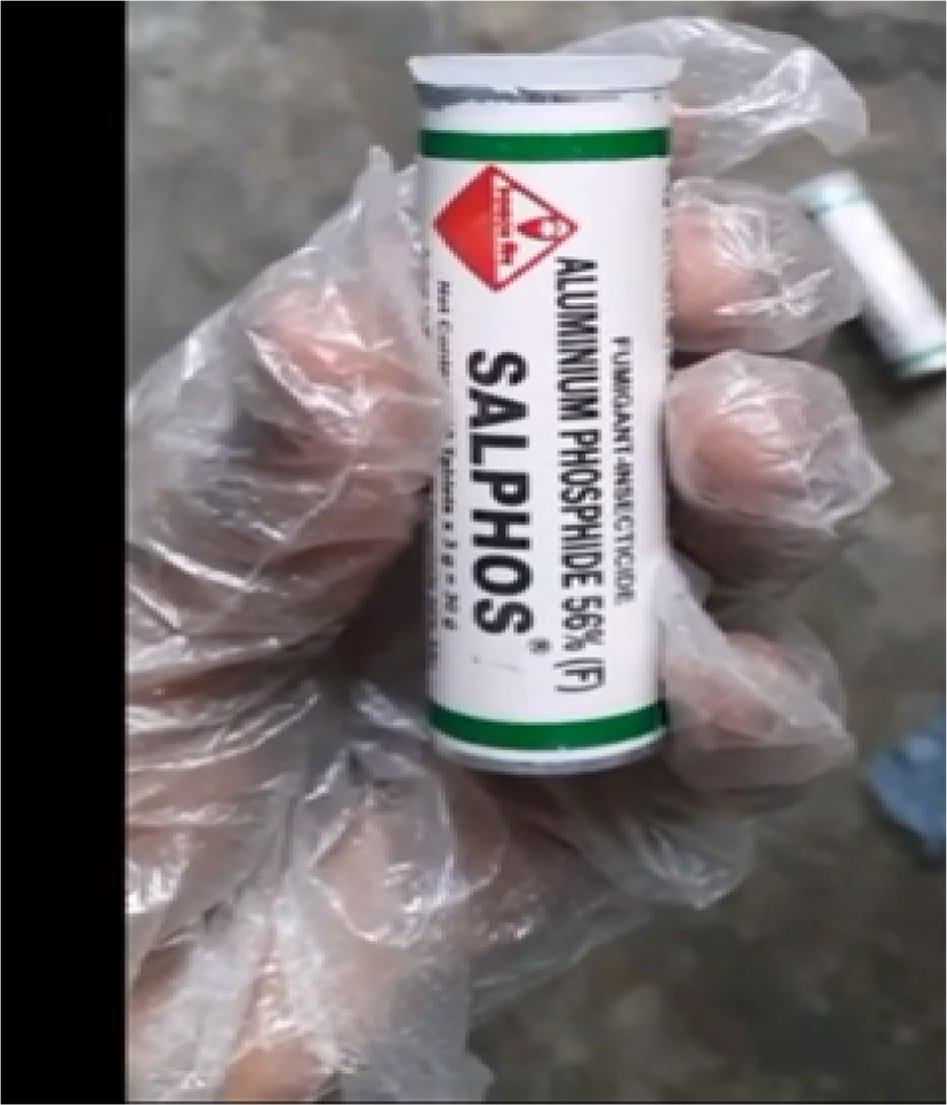
Salphos.

The chickpea fresh seeds locally available were collected. The seeds were sun dried for keeping in presterilized airtight tin containers. For testing in vivo preservative potential method of Kumar^25^ were followed with slight modification. For this mixture oil, salphos (Fig. 1) (Aluminium phosphide) tin containers and polyethylene bags were taken. Tin containers and polyethylene bags farmers are using in this area for chickpea storage. Since mixture oil are in liquid form 200 g of chickpea seeds were taken in 250 ml size tin containers and polyethylene bags. For chickpea seeds treating did preparation of two doses of oil mixture firstly by taking 1000 ppm (0.50 ml) and another 1500 ppm (0.76 ml) in tin containers and polyethylene bags of 250 ml capacity having of 200 g seeds separately. The sterile cotton swabs weighing 0.50 g by soaking of oil doses were wrapped in sterilized muslin cloth weighing of 0.75 g were placed at mid point of each container of chickpea seeds. Similarily, 200 g samples of chickpea seeds were treated with salphos from 1000 ppm (0.50 g) or 1500 ppm (0.76 g) of tablet in 250 ml containers. These were stored in a cabinet for 150 days in the laboratory at room temperature. Each set was having of three replicates. Fungal investigations on seeds were carried out by agar plate technique of Muskett^9^ and the standard blotter technique^10^ of De Tempe (1953). The insects were examined by hand lens.

In negative control sets, the seeds were dressed with requisite amount of acetone in place of the mixture oil (Lavang and sowa leaf oil) and salphos. The tin and polyethylene containers were sealed airtight. These were incubated at room temperature at 75 ± 5% humidity. Observations for presence/absence of mycoflora were made after 25 days observed upto 150 days separately. After 150 days of storage, germination tests were done by taking 100 seeds randomly from each test lot. This then was aseptically placed in presterilized petridishes containing three layers of moistened blotting paper. These were moistened with sterile water at 3 day intervals. All sets were incubated at 28 ± 2 °C in a dark chamber and germination was assessed from the 2nd to 10th day.

## Results and discussion

**Figure 2:**
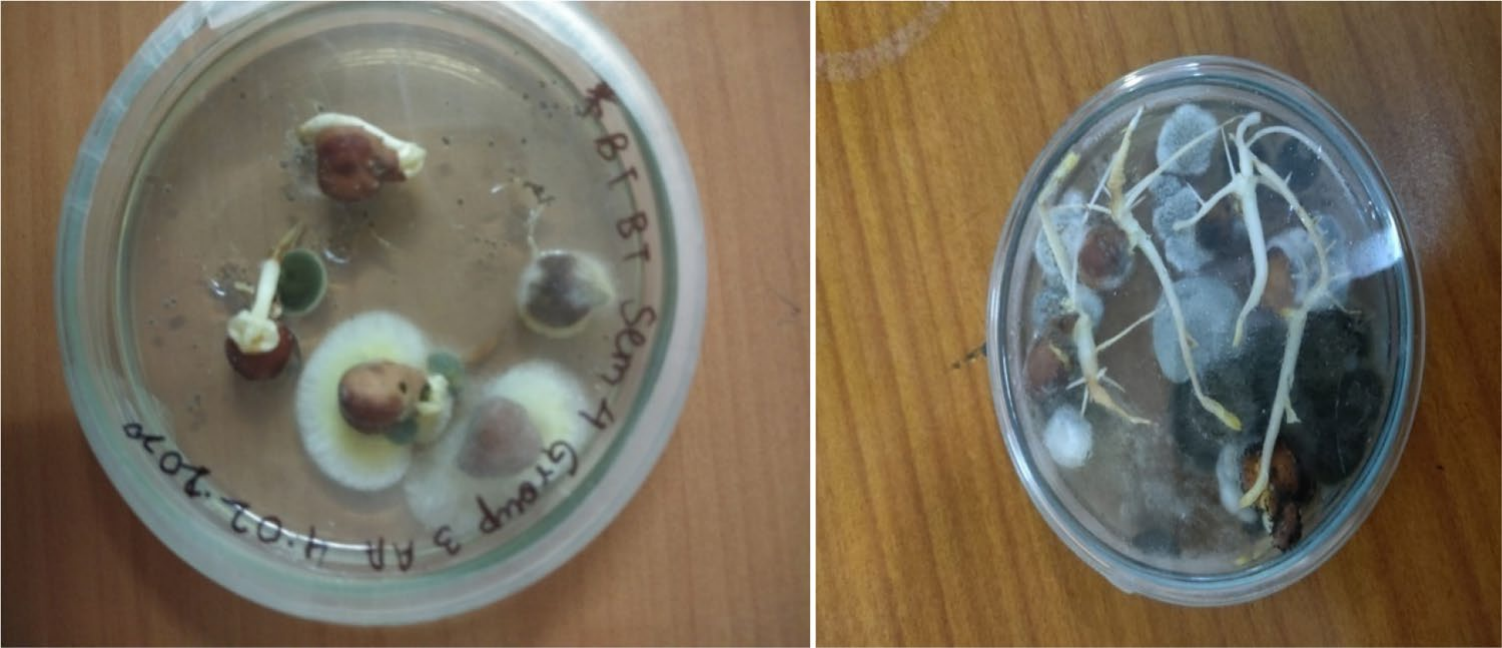
A look at fungi appearing on the chickpea food seeds.

To examine the storage losses, 25 samples of stored chickpea food seeds were collected from grocery stores of Gurgaon and Gorakhpur for examination of associated fungi (Fig. 2) and insects. Seventeen fungal species viz., *Alternaria alternata, Aspergillus flavus, A. niger, A. ochraceous, A. oryzae, A. terreus, A. sydowi, Chaetomium globosum, Colletotrichum dematium, Curvularialunata, Fusarium moniliforme, F. oxysporum, F. solani, Penicillium italicum, Rhizopus stolonifer, Rhizoctonia solani, Sclerotium rolfsii* were found to be associated in stored chickpea (*Cicer arietinum* L.) seeds on the basis of microscopic and macroscopic observations. The agar plate method of study revealed presence of 17 fungi while blotter had 13 fungal species. In these *Aspergillus flavus, A. niger, Fusarium oxysporum* had dominance in terms of per cent occurrence showing 55.3, 50.4, 40.1.0% on blotter and 51.0, 52.0, 50.0% in agar plates, respectively (Table 1)*.*

**Table 1:** Mycological analysis of stored food seeds of chickpea.

Fungi recorded	Blotter method		Agar plate method	
	US	SS	US	SS
*Alternaria alternate* (Fr.) Keissler	4.1 ± 0.05	2.0 ± 0.03	4.1 ± 0.03	–
*Aspergillus flavus* Link	55.3 ± 0.08	20.1 ± 0.09	51.0 ± 0.16	24.9 ± 0.06
*A. niger*van Tieghem	50.4 ± 0.27	23.7 ± 0.08	52.0 ± 0.26	27.7 ± 0.03
*A. ochraceous* Wilhelm	13.0 ± 0.17	4.5 ± 0.03	12.0 ± 0.25	6.7 ± 0.13
*A.oryzae* (Ahlburg) E. Cohn	3.0 ± 0.06	–	3.1 ± 0.05	–
*A. terreus*Thom	11.0 ± 0.17	3.5 ± 0.17	20.6 ± 0.27	5.7 ± 0.17
*A. sydowi* (Bainier and Sartory) Thom and Church	4.3 ± 0.07	2.1 ± 0.06	6.0 ± 0.05	1.0 ± 0.04
*Chaetomium globosum* Kunze	–	–	16.0 ± 0.05	1.0 ± 0.04
*Colletotrichum dematium* (Pers.) Grove	–	–	6.0 ± 0.04	1.0 ± 0.03
*Curvularia lunata* (Wakker) Boedijn	4.1 ± 0.05	2.1 ± 0.04	6.0 ± 0.03	1.0 ± 0.04
*Fusarium moniliforme* Sheldon	3.2 ± 0.01	3.0 ± 0.02	4.1 ± 0.09	–
*F. oxysporum* von Schlechtendal	40.1 ± 0.31	17.6 ± 0.26	50.0 ± 0.31	27.7 ± 0.25
*F. solani* (Mart.) Sacc	2.1 ± 0.04	2.3 ± 0.06	3.1 ± 0.05	1.3 ± 0.05
*Penicillium italicum* Wehmer	–	–	2.2 ± 0.04	–
*Rhizopus stolonifer* Vuillemin	3.3 ± 0.03	–	0.2 ± 0.01	–
*Rhizoctonia solani* J.G. Kühn	4.2 ± 0.07	2.1 ± 0.06	6.0 ± 0.05	1.0 ± 0.04
*Sclerotium rolfsii* Sacc	–	–	6.2 ± 0.03	2.0 ± 0.03

*US* unsterilized seeds, *SS* sterilized seeds, *SD* standard deviation.

–Absence of fungal species.

*Values given are mean of three replicates.

**Figure 3:**
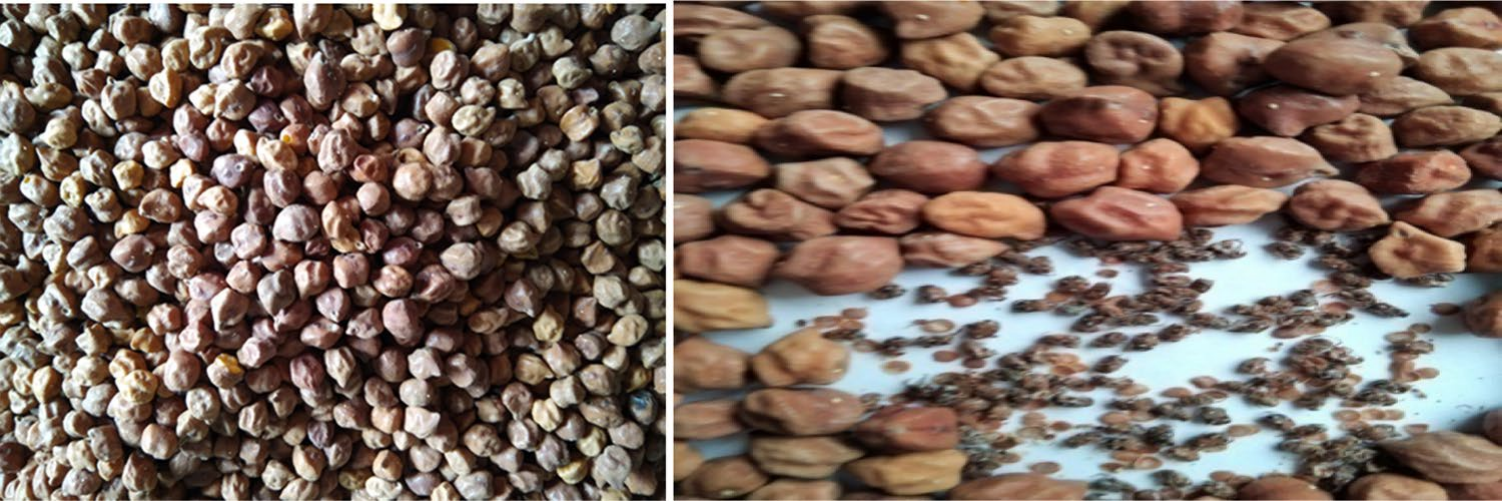
A look on chickpea food seed deterioration by insect.

The insect analysis revealed presence of only one species of Bruchid (*Callosobruchus chinensis* L.) in all the 25 samples of stored chickpea food seeds (Fig. 3). The adult beetle is chockalate coloured, oval shaped, 3–4 mm long. The body is pointed towards front. More attacks were observed during July–August in the stores. This showed feeding by making a small hole. There was presence of white eggs in Infested seeds on the surface and round exit holes with the 'flap' of seed coat.

Time to time fungal species were observed by various workers viz., Nine fungal species -*Alternaria alternata*, *Aspergillus flavus*, *Aspergillus niger*, *Curvularia lunata* [*Cochliobolus lunatus*], *Fusarium moniliforme* [*Gibberella moniliformis*], *Helminthosporium sativum* [*Cochliobolus sativum*], *Mucor* sp., *Penicillium notatum* and *Rhizopus nigricans* [*R. stolonifer*] fromKumarganj, Faizabad India^26^; thirty fungal species were isolated from these seeds and most abundant-among these, were *Alternaria alternata, Chaetomium* spp., *Penicillium citrinum, Aspergillus niger, A. flavus, Rhizopus nigricans, Fusarium oxysporum*^27^; many fungal species viz. *Alternaria alternata, Aspergillus terrus, A. flavus, A. fumigatus, A. niger, Botrytis* sp., *Cladosporium, Curvularialunata, Fusarium solani, F. moniliforme, F. oxysporum, Macrophominaphaseolina, Penicillium notatum, Rhizoctonia* sp. and *Rhizopus nigricans*^28^; seven fungal species such as *Alternaria alternata, Aspergillus flavus, A. niger, A. fumigatus, Curvularialunata, Fusarium monoliforme and Rhizoctonia solani*^29^; Thirteen different fungi like *Actinomucor repens, Alternaria alternata, Aspergillus flavus, A. fumigatus, A. niger, A. ochraceus, Cladosporium* sp., *Fusarium oxysporum, Fusarium sp., Mucor varians, Penicillium notatum, Phomaherbarum, Rhizopus stolonifer*^30^; Aspergillus niger (83%), Fusarium solani (79%), Aspergillus flavus (75%), *Alternaria alternata* (60%) and *Aspergillus nidulans* (57%). Minimum seed borne fungi were reported to be*Rhizopus stolonifer*, and Fusarium oxysporum^31^; 14 fungi comprising 12 genera namely *Aspergillus flavus, Aspergillus niger*, *Aspergillus* sp, *Botryodiplodiatheobromae*, *Botryti cinerea*, *Curvularialunata*, *Chaetomium globosum, Cladosporium* sp., *Colletotrichum dematium, Fusarium oxysporum*, *Macrophomina phasaelina*, *Rhizoctonia solani*, *Rhizopus stolonifer* and *Stemphylium sarciniforme*^32^; Nine fungal species namely Alternaria alternata (Fr.) Keissl., *Aspergillus flavus* Link, *Aspergillus niger* van Tieghem, *Aspergillus oryzae* (Ahlburg) E. Cohn, *Fusarium oxysporum*Schlecht. Emend. Snyder & Hansen, *Macrophominaphaseolina* (Tassi) Goid*., Mucor* sp., *Penicillium italicum*Wehmer and *Sclerotium rolfsii* Sacc^33^, *A. flavus, A. fumigatus and A. niger, Drechslera tetramera, Fusarium moniliforme, Rhizopus stolonifer*^34^. The fungal species viz*., Chaetomium globosum, Colletotrichum dematium, Penicilliumitalicum, Sclerotium rolfsii *(could) did not grow on *blotters. A* study^26^ revealed that *P. notatum* developed on agar plate only^26^. This may be because of fungal nutritional requirements. The no of fungal species were reduced in surface sterilized seeds which indicates that many of the fungi were located on seed coat. The sterile chick pea seeds with 0.1% Mercuric chloride projected less per cent occurrence of fungal colonies (Table 1).

The seed mycoflora devalue the seed quality, reduce its nutritional value and cause a germination failure of the seedlings and of the crop raised from such infected seeds^27^. Based on the data in the Table 2, *A. flavus, A. niger, F. oxysporum* and the insect species—*C. chinensis* played an important role in weight loss, seed germination, carbohydrate and protein content. *A. flavus* inoculated seeds showed 50%, *A. niger* 53%, *F. oxysporum* 40% while insect inoculated 39% while control set without fungus or insect inoculation showed 90–98% seed germination. The seeds inoculated with fungi/insects showed 30.1, 29.4, 28.7, 27.4% carbohydrate content while control set showed 61%. It is evident from Table 2 that fungal/insect inoculated chickpea seeds after 21 days of storage had 10.3, 9.3, 10.4, 8.5% protein content while control seeds were having 20% protein content. On account of wide occurrence and their pathogenicity these were selected as test organisms.

**Table 2:** Evaluation of effect of Fungal/insect species on chickpea food seeds after 21 days storage.

Fungus/insect species	Weight loss (g)		Germination (%)		Carbohydrate (%)		Protein (%)	
	C	T	C	T	C	T	C	T
*Aspergillus flavus*	–	0.189	95	50	61	30.1	20	10.3
*A. niger*	–	0.183	98	53	61	29.4	20	9.3
*F.oxysporum*	–	0.181	90	40	61	28.7	20	10.4
*Callosobruchus chinensis*	–	0.185	96	39	61	27.4	20	8.5

*C* control, *T* treatment.

–: Nil.

Storage conditions and containers are significant in preservation and maintenance of healthy seed during post harvest period^35^. The variation in fungal species may be due to different isolation periods and different storage containers. The isolated species in present investigation are different may be due to different climatic conditions.

The yield of essential oil by hydrodistillation of Lavang (clove bud) was 13.57% while the leaves of *A. graveolens* L. was 1.05% respectively. Fumigant fungitoxic action study of both oils at 500 ppm (0.025 ml) concentration showed 100%inhibition against *Aspergillus flavus, A. niger, Fusarium oxysporum* separately.

It has been extensively reported that plant based essential oils are alternative to synthetic pesticides since they are known to have minimal environmental impact and danger to consumers^36–38^.

For prescription an appropriate dose for the determination of MIC (minimum inhibitory concentration) is necessary. In the study*, Lavang oil (clove)* displayed highest antifungal activity against *Aspergillus flavus, A. niger, Fusarium oxysporum* and completetely inhibited mycelial growth (100%) of fungal speciesat 300 ppm and found fungicidal at 400 ppm. Sowa oil showed complete inhibition at 400 ppm and found fungicidal at 500 ppm. While mixture oil showed complete inhibition (100%) and fungicidal action at 400 ppm against domiant fungi-*Aspergillus flavus, A. niger, Fusarium oxysporum* (Table 3). Both Lavang (clove) and sowa (dill) oil showed 100 per cent repellent activity at 0.01 ml dose. This was also cidal at 0.02 ml dose against test insect-*Callosobruchus chinensis. M*ixture of both oils was cidal at 0.02 ml dose.

**Table 3:** Minimum inhibition concentration (MIC) of *Lavang, sowa* leaf oil and mixture oil.

Oil concentration (ppm)	Percent inhibition of fungal species by oil/mixture oil								
	Lavang oil			Sowa oil			Mixed Lavang and sowaoils		
	*A. flavus*	*A. niger*	*F. oxysporum*	*A. flavus*	*A. niger*	*F. Oxysporum*	*A. Flavus*	*A. niger*	*F. oxysporum*
100	60 ± 0.11	62 ± 0.10	65 ± 0.10	30 ± 0.10	31 ± 0.11	32 ± 0.12	60 ± 0.10	62 ± 0.10	58 ± 0.11
200	70 ± 0.11	72 ± 0.10	82 ± 0.10	39 ± 0.10	38 ± 0.11	42 ± 0.12	82 ± 0.10	82 ± 0.10	83 ± 0.11
300	100 ± 0.14	100 ± 0.13	100 ± 0.15	70 ± 0.14	75 ± 0.14	72 ± 0.13	92 ± 0.14	91 ± 0.14	93 ± 0.13
400	100* ± 0.21	100* ± 0.22	100* ± 0.23	100 ± 0.21	100 ± 0.20	100 ± 0.22	100* ± 0.21	100* ± 0.22	100* ± 0.21
500	100* ± 0.11	100* ± 0.12	100* ± 0.10	100* ± 0.11	100* ± 0.12	100* ± 0.10	100* ± 0.12	100* ± 0.11	100* ± 0.13
600	100* ± 0.08	100* ± 0.07	100* ± 0.09	100* ± 0.07	100* ± 0.06	100* ± 0.09	100* ± 0.08	100* ± 0.07	100* ± 0.09

*SD* standard deviation.

*Fungicidal; *Values given are mean of three replicates.

The study on mixture of oils for antifungal spectrum revealed it knocked all seventeen fungi (*A. alternata, Aspergillus flavus, A. niger, A. ochraceous, A. oryzae, A. terreus, A. sydowi, Chaetomium globosum, Colletotrichum dematium, Curvularia lunata, Fusarium moniliforme, F. oxysporum, F. solani, Penicillium italicum, Rhizopus stolonifer, Rhizoctonia solani, Sclerotium rolfsii*) at 500 ppm concentration. It showed 70–93% inhibitory activity against all 17 fungi at 300 ppm isolated from chickpea seeds (Table 4). The oil mixture’s activity was not affected by autoclaving at 15 lb/psi at 120 °C. It (safely) inhibited fungal growth even after storage of oil upto150 days (Table 5).

**Table 4:** Fungitoxic spectrum of mixture oil (Lavang and sowa leaf oil in 1:1 ratio) at different concentrations against fungi isolated from stored chickpea food seeds.

Fungal species	Per cent inhibition of mycelial growth of isolated fungi (%)		
	300 ppm	500 ppm	700 ppm
*Alternaria alternata*	74.1 ± 0.10	100.3 ± 0.11	100.0 ± 0.12
*Aspergillus flavus*	92 ± 0.14	100 ± 0.12	100 ± 0.11
*A. niger*	91 ± 0.14	100.0 ± 0.16	100 ± 0.14
*A. ochraceous*	84.4 ± 0.13	100.0 ± 0.13	100.0 ± 0.12
*A. oryzae*	71.1 ± 0.23	100.0 ± 0.16	100.0 ± 0.18
*A. terreus*	77.7 ± 0.12	100.0 ± 0.16	100.0 ± 0.07
*A. sydowi*	70.1 ± 0.13	100.0 ± 0.16	100.0 ± 0.21
*Chaetomium globosum*	85.1 ± 0.19	100.0 ± 0.12	100.0 ± 0.17
*Colletotrichum dematium*	77.1 ± 0.10	100.0 ± 0.12	100.0 ± 0.13
*Curvularialunata*	74.1 ± 0.14	100.0 ± 0.15	100.0 ± 0.18
*Fusarium moniliforme*	71.0 ± 0.23	100.0 ± 0.14	100.0 ± 0.19
*F. oxysporum*	93 ± 0.13	100 ± 0.13	100.0 ± 0.23
*F. solani*	83.1 ± 0.13	100.0 ± 0.15	100.0 ± 0.16
*Penicillium italicum*	84.4 ± 0.10	100.0 ± 0.13	94.3 ± 0.14
*Rhizopus stolonifer*	74.1 ± 0.11	100.2 ± 0.12	100.0 ± 0.14
*Rhizoctonia solani*	84.1 ± 0.10	100.0 ± 0.13	100.0 ± 0.14
*Sclerotium rolfsii*	87.1 ± 0.10	100.0 ± 0.16	100.0 ± 0.17

*SD* standard deviation.

*Values given are mean of three replicates.

**Table 5:** Effect of physical factors on the fungi toxicity of mixture oil (Lavang and sowa leaf oil).

Applied physical factors	Per cent inhibition of mycelial (%) growth at its MIC
Autoclaving (15lbs/psi at 120 °C) For 15 min	100 ± 0.19
**Storage time(in days)**	
25	100 ± 0.11
50	100 ± 0.13
75	100 ± 0.14
100	100 ± 0.22
125	100 ± 0.21
150	100 ± 0.23

*SD* standard deviation.

*Values given are mean of three replicates.

In this study, *Lavang oil* showed MIC 300 ppm, Sowa oil 400 ppm while the mixture had 400 ppm against *Aspergillus flavus, A. niger, Fusarium oxysporum. *Previous studies depicted that there is a marked variation in the MIC of different plant oils at different concentrations against *A. niger *viz*., Ocimum adscendens*at 200 ppm^39^ *Syzygiumaromaticum* (L.) Merrilland Perry 200 ppm^40^; *Cedrus deodara* at 1000 ppm and *Trachyspermum ammi* at 500 ppm^41^; *Adhatoda vasica*at 500 ppm^42^. C*uminum cyminum* at 400 ppm^25^; *Tinospora cordifolia* at 400 ppm^43^. The differences in the MIC of various plant oils may be due to their being different in chemical constituents or other chemical or physical parameters.

A fungicide must retain its fungitoxicity even after autoclaving^44^. The fungitoxicity of oil mixture (Lavang and sowa leaf oil) retained the fungitoxicity (Table 4) even after autoclaving (15lbs/psi) like that of *Ageratum conyzoides*^45^; *Nardostachys jatamansi*^46^; *Adhatodavasica* oil^42^; *Cuminum cyminum*^25^ and *Tinospora cordifolia*^47^. This quality of oil will facilitate the isolation of their constituents in active state. It is also evident that a fungicide must be able to retain its activity for a long storage period. The fungitoxic factor in the oil of *Adenocalyma allicea* was lost within 21 day of storage^48^ while retained for longer periods in the oils of *Ageratum conyzoides*^45^; *Trachyspermum ammi*^41^); *Adhatoda vasica*^42^* Cuminum cyminum*^25^, *Tinospora cordifolia*^47^, *Lobularia maritime*^49^, *Cinnamomum cassia*^50^. The antifungal Toxicity of mixture oil (Lavang and sowa leaf oil) was not affected by storage upto 150 days. It may be suggested that the oil mixture (Lavang and sowa leaf oil) can be safely stored for long periods (150 days) without having any adverse effect on toxicity.

The physiochemical properties of the Lavang and sowa leaf oil are recorded in Table6. GC–MS analysis of Lavang oil revealed major components viz., 75.63%eugenol, 12.67%caryophyllene, 8.34% eugenyl acetate and 1.63% α-humulene while the minor components were 0.03% β-elemene, 0.06% α-cadinene and 0.07% ledol. The GC–MS analysis of sowa leaf oil showed presence of major constituents viz., 25.14% apiole, 12.79% o-cymene, 15.67% α-thujene, 7.13% β-phellandrene while minor components viz., 4.87% exo-2-hydroxycineol, 4.13% limonene, 2.83% 3-isoproyl-4-methyl-1-pentyn-3-ol, 2.14% myristicine (2.16%) and 2.13% dithydroumbell the oil ulone.

**Table 6:** Physico-chemical characteristics of extracted oil through hydrodistillation.

Name of oil	Specific gravity	Refractive index	Optical rotation	Acid value	Ester value	Saponification value	Solubility characteristic
Lavang (bud) oil	1.053 ± 0.0010	1.539 ± 0.0013	1°64′ ± 1°10′	3.941 ± 0.21	37.89 ± 0.30	41.09 ± 2.1	Found soluble in 2 volume of 70% alcohol
Sowa leaf oil	0.9233 ± 0.0012	1.4606 ± 0.0002	+ 9.342 ± 2.0	15.64 ± 0.12	13.5 ± 0.12	182.25 ± 2.3	Insoluble in water, but miscible in alcohol on warming

*SD* standard deviation.

*Values given are mean of three replicates.

The study also showed that mixture oil (Lavang and sowa leaf oil) was more effective than salphos. It is evident from Table 7, that salphos controlled a maximum of 3 fungi while the oil mixture showed complete seed protection showing growth of no fungi and insect-*Callosobruchus chinensis* even after 150 days of storage. It shows its potential efficacy as the Seed Protectant as it is able to protect all 17 fungi (Table 7). Hence it appears that mixture oil (Lavang and sowa leaf oil) increases the chickpea seeds shelf life. The seeds which served as control, showed proliferation of all 17 fungal species after 150 days of storage. Our study revealed that mixture oil (Lavang and sowa leaf oil) was more fungitoxic than salphos used in our experiments. No insect species was observed in the oil mixture treated seeds in both containers while it was present in the sulphos treated and control sets. The antifungal and insect repellent propertyof this mixture oil (Lavang and sowa leaf oil) suggests to exploit it as an ideal protectant of chickpea seeds during storage.

**Table 7:** Mycological analysis of 150 days stored chickpea seeds treated with mixture oil (Lavang and sowa leaf oil) and salphos.

Fungi recorded	Mixture oil		Salphos	
	US	SS	US	SS
*Alternaria alternata*	–	–	5.1 ± 0.07	–
*Aspergillus flavus*	–	–	16.1 ± 0.04	–
*A. niger*	–	–	17.0 ± 0.13	4.5 ± 0.11
*A. ochraceous*	–	–	19.0 ± 0.21	3.7 ± 0.04
*A.oryzae*	–	–	9.0 ± 0.01	4.5 ± 0.05
*A. terreus*	–	–	–	–
*A. sydowi*	–	–	7.6 ± 0.12	2.7 ± 0.08
*Chaetomium globosum*	–	–	14.7 ± 0.07	1.5 ± 0.01
*Colletotrichum dematium*	–	–	–	–
*Curvularia lunata*	–	–	1.0 ± 0.24	5.4 ± 0.07
*Fusarium moniliforme*	–	–	–	–
*F. oxysporum*	–	–	11.3 ± 0.02	1.0 ± 0.07
*F. solani*	–	–	8.3 ± 0.11	0.3 ± 0.06
*Penicillium italicum*	–	–	7.3 ± 0.11	1.3 ± 0.06
*Rhizopus stolonifer*	–	–	5.1 ± 0.04	–
*Rhizoctonia solani*	–	–	9.0 ± 0.01	0.5 ± 0.01
*Sclerotium rolfsii*	–	–	9.3 ± 0.11	0.7 ± 0.03

*US* unsterilized seeds, *SS* sterilized seeds, *SD* standard deviation.

–: Absence of fungal species.

*Values given are mean of three replicates.

After 150 days of storage, germination tests revealed 90–95% seed germination in oils mixture’s treated sets, salphos treated sets revealed 60–65% while control set revealed 50–55% seed germination from seeds of both containers. The mixture oil showed no adverse effect on seedling growth and general health and morphology of plants. Thus, the mixture oil (Lavang and sowa leaf oil) showed great potential as protective agent for chickpea seeds against spoilage by fungi and insects during storage.

## Conclusion

Our study revealed that oilmixture (Lavang (clove) and sowa (dill) leaf oil) was more fungitoxic than tested synthetic pesticides-Salphos. It completely protected from Bruchid (*Callosobruchus chinensis*). This indicates its possibility of its exploitation in the form of seed protectant of chickpea food seedsfor storage. Lavang and sowa is an indigenous plant. It grows luxuriantly. Essential oil can be easily made available upon extraction and formulations can be prepared. It will be a renewable source of fungitoxicantand bruchid repellent which will be keeping chickpea food seeds without damage. There will be protection of edible food by edible oils without any toxic effect on human and environment will remain safe. This study would provide an useful information to readers for protecting food seeds by edible ones.
